# Water: Its History, Characteristics, Hygienic, and Therapeutic Uses

**Published:** 1861-07

**Authors:** Samuel W. Francis


WATER: ITS HISTORY, CHARACTERISTICS, HYGIENIC, AND THERAPEUTIC USES.—By *Samuel W. Francis, A. M., M. D.* —No. 1.—*Religious History.*—When the pleasing duty is assigned us of discussing a subject which could well employ the thoughtful mind for many busy years, we enter upon a consideration of its beauty and depths with a calm pensiveness, and pursue its charms with a quiet enjoyment, unlimited by the narrow bounds of a set task. We feel that humanity is interested in the same object of our investigation, and appreciate, to the fullest extent, the contemplation of that which is common, in its usefulness, to all, and may, with impunity, be regarded by mankind as the only true democratic link in nature.

Water, as a theme, is as expansive as the ocean—as endless, in its teachings of philosophy, as the globe itself—and as truthful in its reflections as it is pure in its organization: as an inhabitant of the earth, one of the oldest—as a parent of secondary growths, the most antiquated. We find recorded in the pages of Holy Writ:—"Darkness was upon the face of the *deep*," and "God moved on the face of the *waters*." (Gen. i., 2.) It was not until the waters were formed that the firmament (heavens) was made. The waters existed before even dry land appeared. When that grand scheme of nature had been fulfilled—when all the plants of the earth, and lights, had been created, to balance the laws of the sphere —there was a stillness, such as never can be equaled again.

Then, on the fifth day, "God said, let the *waters* bring forth abundantly the moving creatures that hath life;" not only "whales and every living thing which the waters brought forth after his kind," but "the fowl that may fly above the earth in the open firmament of heaven."

In the second chapter of Genesis, we find that when all was completed and God rested from his labors, and was well pleased that it was very good, " there went up a mist from the earth and watered the whole face of the ground."

The garden of Eden—the paradise of man before his fall—was well watered by a stream that parted into four heads on emerging from its beautiful seclusion, where man was, for centuries, to dwell in purity and innocence. It is important to be thus minute in detailing the smallest facts connected with the earlier history of water in the beginning of the world—for it establishes an interesting and instructive chain of evidence from chaos to the end of the slow-paced movements of advancing time, that at once stamps upon the face of a truthful record the priority of water. And what can be of greater moment to man than this mundane sphere while he lives and breathes and moves? And when he learns of the part borne by water at the commencement of all, he experiences additional interest in tracing out its meanderings through the green pastures. Leaving a large margin in the book of life, he passes on to meditate upon it as the key-note of all that breathes.

To touch briefly upon the uses made of water in the Bible for the benefit of man, we find many instances of historical importance, involving moral teachings, miraculous cures, and suggestive lessons. When the world had abandoned itself to sin, and mankind refused to listen to the admonitions of a merciful God, the elements bore down the messages of a wrathful indignation from the fountain-head of Truth, and thousands were thus swallowed up in water. The Deluge washed all from the record of humanity, save those who, exhibiting a pious faith, had gathered themselves together in the Ark of the Covenant, the sole representatives of this future world; and that the human mind might not lose its just ambition in ages yet to come, Iris appeared, spanning the heavens and telling, in all the softening colors of prismatic truth, the mercy of an injured Deity. Strange, indeed, does it appear to the contemplative mind, that, while the world was first destroyed by water, its second end will be produced by want of it!

The wells of Jacob are still in existence, and may be seen by the traveler, or met with by the weary pilgrim on his lonely path of duty and of sorrow.

While all the males in Egypt were being slain to restrict the increase of the Israelites; while parents wept and mothers sat bereaved of their bright future prospects, the infant Moses was placed upon the bosom of the placid stream to be wafted at the disposition of the God of Israel, the future leader of a mighty host, the faithful ruler of an ungrateful people. Borne along the banks of Pharaoh's river, he was rescued by the heathen. Thus an eventful era in the history of the world turned upon the fated voyage of the little fragile bark, and the sleeping innocent, the embryo of future purposes, the instrument of Providence. When the seven plagues failed to soften Pharaoh's heart, and he repented him of his rash permit to depart, though many miracles had been performed to demonstrate the existence of a mightier power, it remained for the Red Sea to roll back at the word of the all-powerful JAH, to liberate forever the bondsmen from the tyrant's sway. Returning, at the second mandate, to its former bed, it swallowed up the worshiper of idols, with his great company of soldiers, and left another mark in the records of the nation's progress that foretold a change of customs, habits, and religion.

The children of the wilderness journeyed on, obedient to the movements of the cloudy pillar; and when at length surrounded by a desert waste, with no appreciable evidences of relief from thirst and hunger, they doubted the wisdom of an ever mindful guide, and with parched lips, and uplifted eyes, rebuked the Omnipotent for such painful trials, the flinty rock was smitten, and pure water gushed forth to alleviate their sufferings and confirm their faith.

The miraculous adventures of the prophet Jonah form one of the most interesting facts connected with the earlier periods marked in the writings of the Old Testament. Elijah, when there had been few before him to exhibit signs of faith and reliance on the aid of Heaven, fearlessly crossed the river Jordan, with no power save that of confidence in God. And when Naaman had tried all the remedies of his own dominion, the simple answer to his inquiry for beneficial means to be employed, was but to bathe just seven times in the river Jordan. The keeping back the rain for many days, and then pouring down a plentiful supply on dried up lands, in answer to the Prophet's prayer, serve to indicate the mighty influence upon the dullest intellects or hardest hearts, produced by droughts, the consequences of controlling nature's plans.

When Hagar was abandoned, with her child, to the care of nought save the proud elements, and fainting, they sat down to die for want of sustenance, an angel of the Lord appeared and pointed out the spring of cool, refreshing water. David exercised more praiseworthy self-control when refusing the helmet of water, purchased by the blood of a few, than when leading his victorious armies forth to meet a valiant enemy. How suggestive of quiet benefits and soothing influences are the pools of Siloam and Bethesda! How surpassingly beautiful is the story of the angel descending from above to purify the water and medicate the stream, where old and indigent, decrepid and enfeebled human brothers laved their wounds and found a panacea.

More of the miracles of our Savior were performed on the water than we can find recorded of any other subject of Divine regard. The tribute money, demanded from his followers, was instantly obtained by casting forth a hook and bringing up a fish whose belly contained the money looked for. The miraculous draught of fishes strengthened much the faith of the disciples, themselves fishermen. The walking on the water, amid the shades of evening, called forth a corresponding confidence in Peter, and excited wonder and amazement. It was a storm at sea, threatening every instant to engulf the hardy seamen, that brought forth the piteous bewail, "Save Lord or we perish," and received that mighty, powerful, yet expressive response and edict to the winds and waves:

"Peace, be still!"

How often, when the eager multitude had flocked to listen to their beloved Master, did he preach to one and all from the side of some small craft, rendering the vessel as sacred, for his purposes, as the pulpit is in consecrated times. In the Book of Psalms how expressive of purity, holiness, and quiet serenity of existence, is the descriptive phrase: "There is a river, the streams whereof make glad the City of God." Again, we have the beautiful simile, full of force, yet indicative of the gentler emotions of a subdued spirit, the psalmist exclaims, with all the poetry and pathos of one sensible of his condition: "As the hart panteth after the water brooks, so panteth my soul after thee, O God." And a little further on he states, with emphatic zeal, yet abiding self-consciousness of his desolation: "Deep calleth unto deep at the noise of thy water-spouts: all thy waves and thy billows are gone over me."

Innumerable are the metaphorical allusions to water in the Holy Bible, as evincing hope, sorrow, joy, faith, and repentance. And, indeed, one of the greatest examples of humility recorded in the New Testament is when our Savior knelt down at the side of his disciples, to wash their feet; symbolical of purifying their souls, and humbling their views of a Christian's superiority over sublunary offices.

It is also remarkable, and worthy of note, that the only time our Savior wrote was on the sand by the sea shore. The first great miracle performed was at the marriage feast of Cana, where " the conscious water saw its God and blushed."

When David, inspired by a sense of duty and a holy love of what was right, went forth to arm himself against the mighty Goliath, leader of the Phillistines, he refused to wear the proffered gift of armor and the richly-ornamented apparel of his own great king; and, walking down, chose him five smooth stones out of the brook, which served him, aided by Divine assistance, to overcome the titanic warrior, and free his country from a warlike foe.

When an invitation is given to the lost sinner, and he is urged to move on towards the Throne of Grace, we find the following: " Ho! every one that thirsteth, come ye to the living waters."—Thirst being taken as the greatest suffering to be endured by man while in a state of unregeneration. It is not let him that is an hungered, eat! But ye that are wearied; that have exhausted all the energies of matter; who have not vital force to overcome surrounding objects; who need refreshing comfort; who desire what can alone prove soothing and salutary to the wasted spirit; come to the Fountain of Life and *drink.*

And still again, as a weapon of punishment, or as a proof of Christian rectitude, do we read the following counsel given to the man of probity when injured by his brother: " If thine enemy *thirst*, give him drink; for in so doing thou shalt heap coals of fire on his head." Here is another pure and holy precept for the follower of true charity: " Whosoever shall give a cup of cold water to one of these, in my name, shall have his reward." And I would ask what passage in the Scriptures is employed to exhibit the suffering in a future world, with a more comprehensive meaning, or a more dreadful warning, than that which speaks of the punishment of Dives while in hell? What did he ask for? What did he desire? What was the sole remaining means of satisfying his cravings for relief from agony untold? That Lazarus might but touch the tip of his tongue with a drop of cold water! The heat to be endured; the living fires of a guilty conscience, corroding with a quenchless remorse; the burning despair of a hopeless misery; all conspired to call forth the solitary desire now left to the doomed man—water!

When the Israelites were in captivity, and hope seemed to vanish for the want of encouraging prospects; their bright features dimmed by the shadows of a heathen bondage: "By the rivers of Babylon they sat down and wept," hanging their harps on aged willows as they drooped in prophetic silence over the murmuring streams.

It is a most interesting fact, suggested to the meditative mind, that while man has been permitted to discover, promulgate and bring to light new truths, invent a thousand wonderful improvements, and unfold a world of scientific paradoxes, all of which is but the laying bare to view the latent treasures always to be found in nature, God built the first vessel that was to surmount the waters of the deep and rescue fallen man from total ruin. How expressive and how true and full of calm, ennobling sentiments are the words of the wisest man that ever lived: "Cast thy bread upon the waters; for thou shalt find it after many days!" (Ecc. xi, 1.)

So great a reverence is felt for water by some heathen nations that it is esteemed a god, and worshiped by them as the ruler of their destinies. Its quiet meanderings through unfrequented regions; its ubiquity; whether it has for its residence the parks of regal splendor, its banks adorned with plants of choicest rarity, or flowing in a gentle, gurgling stream let through the peasant's meadow, refreshing his cattle, beautifying the scenery, reflecting the heavens, and then running on to point man to its sources of purity and irresistible powers. One and all of these attributes seem to have inspired in the bosom of the swarthy Brahmin a feeling of adoration, fear, love, and awe. By the inhabitants of India the river Ganges is regarded as worthy of veneration, and even propitiation. This same river Ganges, which is one of the four parted from the original stream flowing through the garden of Paradise, is worshiped as the nail of the great toe of the god Vishnu.* The blind, infatuated natives seek to wash away their crimes by offering up annually, as an atone-

* Rev. Dr. Scudder, missionary to India.

ment, hundreds of their innocent babes to this insatiable deity. A religion resembling more the fearful demon spoken of in Vattek* than such as one could learn to follow as the means of reaching future joys, in no way corresponding to the laws of true inspiration coming from divine associations.

In years gone by, mermaids were looked upon by seamen with fear and trembling, alike for their fascinating powers and relentless exactions, dwelling in mysterious caverns and subaqueous abodes, the constant resorts of sirens, and other equally unknown agents in behalf of some all-powerful being.

The Indian warrior has often bowed before the throne of the Great Spirit at Niagara, listening to his thunderings as unto some unknown language. If not correct or orthodox, it shows at least how sublime a thought for the ignorant savage, who seeks the future happy hunting grounds as the reward of honest dealings in this world.—*Med. and Surg. Reporter.*

(To be continued.)

* Vattek, by William Beckford, of England.

